# Hypocalcaemia and hospitalization after initiation of bone-modifying agents in dialysis patients

**DOI:** 10.1093/ckj/sfag364

**Published:** 2026-11-02

**Authors:** Takahiro Imaizumi, Nobuhiro Nishibori, Masaki Okazaki, Koya Nagase, Yuko Asano, Yoshihisa Takada, Tomoko Handa, Daisuke Ito, Atsushi Hashizume, Shoichi Maruyama

**Affiliations:** Department of Nephrology, Nagoya University Graduate School of Medicine, Nagoya, Japan; Department of Clinical Research Education, Nagoya University Graduate School of Medicine, Nagoya, Japan; Department of Nephrology, Nagoya University Graduate School of Medicine, Nagoya, Japan; Department of Nephrology, Nagoya University Graduate School of Medicine, Nagoya, Japan; Department of Clinical Research Education, Nagoya University Graduate School of Medicine, Nagoya, Japan; Department of Nephrology, Shinseikai Dai-ichi Hospital, Nagoya, Japan; Department of Nephrology, Nagoya University Graduate School of Medicine, Nagoya, Japan; Department of Nephrology, Nagoya University Graduate School of Medicine, Nagoya, Japan; Department of Clinical Research Education, Nagoya University Graduate School of Medicine, Nagoya, Japan; Department of Clinical Research Education, Nagoya University Graduate School of Medicine, Nagoya, Japan; Department of Clinical Research Education, Nagoya University Graduate School of Medicine, Nagoya, Japan; Department of Clinical Research Education, Nagoya University Graduate School of Medicine, Nagoya, Japan; Department of Clinical Research Education, Nagoya University Graduate School of Medicine, Nagoya, Japan; Department of Advanced Medicine, Nagoya University Hospital, Nagoya, Japan; Department of Nephrology, Nagoya University Graduate School of Medicine, Nagoya, Japan

**Keywords:** bone-modifying agents, dialysis, hospitalization, hypocalcaemia, secondary hyperparathyroidism

## Abstract

**Background and hypothesis:**

Bone-modifying agents are associated with hypocalcaemia in dialysis patients, leading to serious adverse consequences. We examined factors associated with all-cause hospitalization, longitudinal serum calcium trajectories by medication class, and baseline predictors of hypocalcaemia in dialysis patients initiating bone-modifying agents.

**Methods:**

Using the JMDC hospital database, we identified dialysis patients newly prescribed bisphosphonates, denosumab, teriparatide, or romosozumab. Factors associated with all-cause hospitalization were examined using Cox regression. Serum calcium trajectories were assessed over 90 days before and after treatment initiation. Factors associated with hypocalcaemia (corrected calcium <8.0 mg/dl) within 90 days were analysed.

**Results:**

Among 2020 patients on dialysis, 1057 (52.3%) received bisphosphonates; 693 (34.3%), denosumab; 136 (6.7%), teriparatide; and 134 (6.6%), romosozumab. Hospitalization occurred in 425 patients, and recent hospitalization and pretreatment lower corrected serum calcium levels were associated with hospitalization, with no significant differences across medications. Among 374 patients with laboratory data, post-treatment hypocalcaemia occurred in 99 (26.5%). Persistent reductions in serum calcium levels were observed after initiating denosumab or romosozumab. Adjusted odds ratios (OR) for post-treatment hypocalcaemia were 13.4 (6.90–26.1) for denosumab and 8.09 (2.20–29.8) for romosozumab. Pretreatment calcium and parathyroid hormone levels were associated with post-treatment hypocalcaemia [adjusted ORs: 0.29 (0.18, 0.49) and 1.59 (1.05, 2.39), respectively].

**Conclusion:**

In dialysis patients, denosumab and romosozumab were associated with a higher risk of post-treatment hypocalcaemia than bisphosphonates, and pretreatment lower calcium was associated with hospitalization. Our findings highlight the importance of optimizing parathyroid hormone management and enhancing calcium monitoring when initiating bone-modifying agents.

## Graphical Abstract

**Graphical Abstract:**
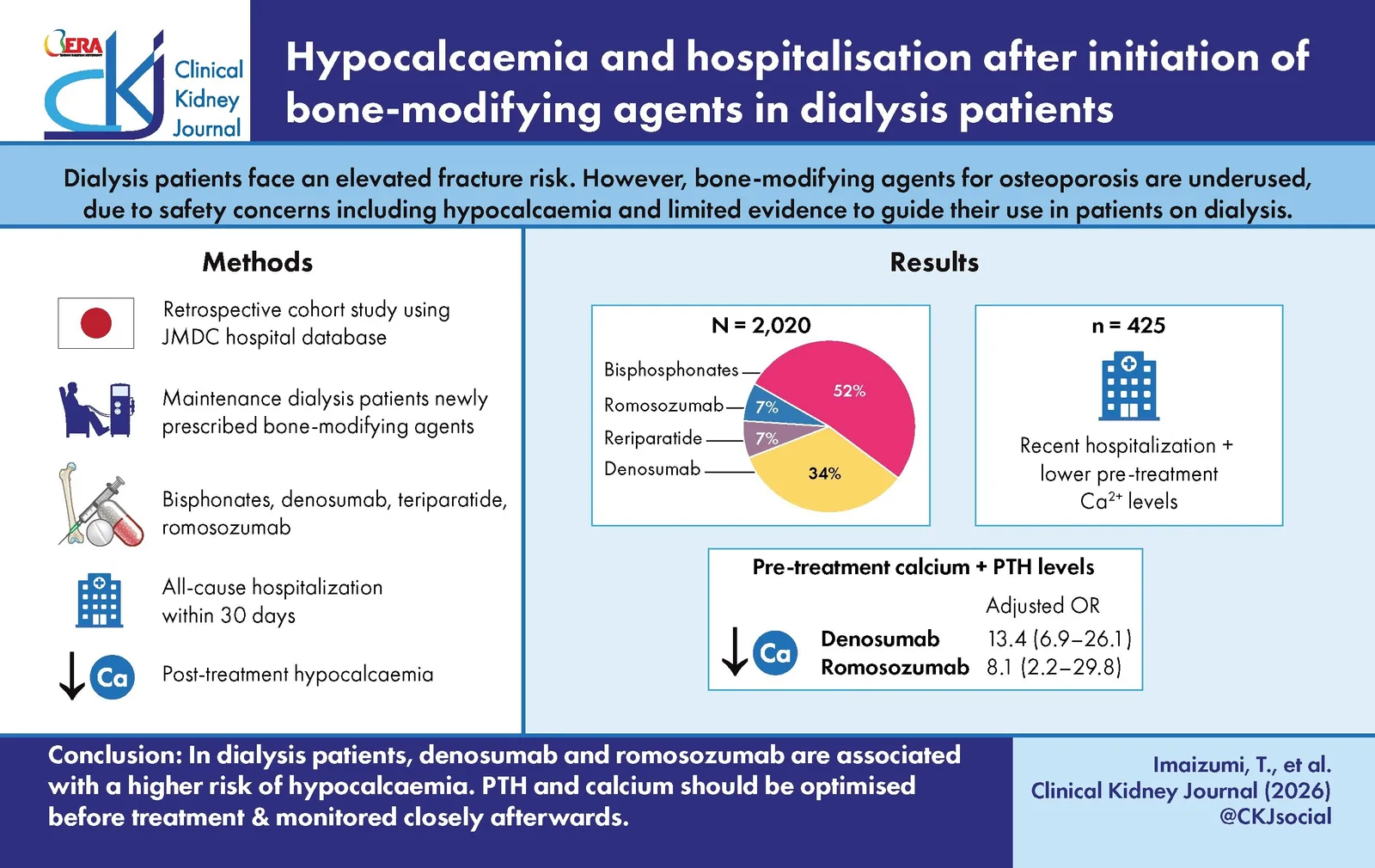

KEY LEARNING POINTS **What was known:**

- Dialysis patients face an elevated fracture risk; however, bone-modifying agents are underused, partly because of hypocalcaemia concerns.
- Denosumab and romosozumab have been associated with hypocalcaemia in case reports and small studies; however, pivotal randomized trials have systematically excluded patients on dialysis, leaving a critical gap in real-world safety evidence.

**This study adds:**

- In a real-world cohort of 2020 maintenance dialysis patients, denosumab and romosozumab were associated with a markedly higher risk of post-treatment hypocalcaemia than bisphosphonates.
- Pretreatment hypocalcaemia was associated with all-cause hospitalization; both pretreatment hypocalcaemia and elevated parathyroid hormone were independently associated with post-treatment hypocalcaemia across medication classes.

**Potential impact:**

- These findings underscore the importance of evaluating serum calcium and parathyroid hormone levels before initiating treatment with bone-modifying agents in patients undergoing dialysis.
- Corrected serum calcium below 9.0 mg/dl may serve as a practical threshold for heightened vigilance, and enhanced calcium monitoring is essential following denosumab or romosozumab initiation in this population.

## INTRODUCTION

Patients receiving maintenance dialysis face a markedly elevated fracture risk, with incidence rates of 12–45 per 1000 patient-years, along with high rates of post-fracture mortality [1–3]. The incidence of hip fractures among haemodialysis patients is ∼five- to six-fold higher than that in the general population [4]. Despite this burden, osteoporosis remains undertreated in dialysis patients; treatment rates are as low as 36% compared with 71% in the earlier stages of chronic kidney disease (CKD) [5], partly due to safety concerns, including hypocalcaemia [6, 7], and compounded by the exclusion of patients with advanced CKD from pivotal randomized controlled trials of bone-modifying agents, resulting in limited evidence to guide clinical decision-making [7].

Management of bone health in dialysis patients is complicated by CKD-mineral and bone disorder (CKD-MBD). Secondary hyperparathyroidism leads to a heterogeneous bone turnover status [8–10], and although bone-modifying agents, such as denosumab and bisphosphonates, are used for fracture prevention [11], their use in dialysis patients carries an increased risk of hypocalcaemia [12–14]. Hypocalcaemia is clinically significant beyond laboratory abnormalities: it may cause neuromuscular manifestations including tetany and seizures, impaire cardiac contractility, and prolong the QT interval, thereby precipitating various events that may require hospitalization. Case reports have documented life-threatening cardiac decompensation following denosumab-induced hypocalcaemia [14–16], and the US Food and Drug Administration added a Boxed Warning citing hospitalization, life-threatening events, and death [17]. However, whether these risks are increased following initiation of bone-modifying agents in real-world settings remains unclear. Parathyroid hormone (PTH) may further modify hypocalcaemia risk and treatment safety [18]. Calcium management in this population requires balancing the risk of hypocalcaemia against that of calcium overload associated with vascular calcification.

Despite the growing awareness of hypocalcaemia as a major adverse event of osteoporosis treatment in patients on dialysis, its temporal course, duration, and variation according to the medication class and baseline PTH status remain poorly characterized. Using a large-scale real-world database, we aimed to (1) identify factors associated with all-cause hospitalization following the initiation of bone-modifying agents, (2) characterize longitudinal serum calcium trajectories by medication class, and (3) identify baseline predictors of hypocalcaemia, with particular attention to PTH status. We additionally examined hypercalcaemia as an exploratory outcome. To our knowledge, this is the first real-world study to simultaneously examine hypocalcaemia risk across multiple bone-modifying agent classes and its association with hospitalization outcomes in maintenance dialysis patients—a population systematically excluded from pivotal randomized trials. The findings may directly inform clinical decision-making regarding calcium and PTH monitoring before and after the initiation of bone-modifying agents in this vulnerable population.

## MATERIALS AND METHODS

### Data source and study population

We used the JMDC database, which contains de-identified hospital-based administrative claims and an electronic health record database covering April 2014 to November 2024, collected from contracted medical institutions across Japan. The details of the database are described elsewhere [19]. We identified patients receiving maintenance dialysis using procedural codes for haemodialysis or peritoneal dialysis recorded in outpatient claims. To ensure stable dialysis status, we required either ≥30 dialysis sessions for haemodialysis or ≥90 days of follow-up with dialysis claims from the first dialysis record.

From this dialysis cohort, we identified patients who newly initiated bone-modifying agents. To ensure new user status, we required a lookback period of at least 6 months from the first dialysis claim to the first bone-modifying agent prescription, during which no bone-modifying agents were prescribed: patients not meeting this requirement were excluded. Eligible medications were classified into four categories: (1) oral and intravenous bisphosphonates (BP), (2) receptor activator of nuclear factor-kappa B ligand (RANKL) inhibitors (denosumab, DMAB), (3) PTH analogues: teriparatide and its derivatives (TPTD), and (4) romosozumab (ROMO): anti-sclerostin antibody (Supplementary Table S1).

The index date (Day 0) was defined as the date on which the first bone-modifying agent prescription met the new user criteria. Patients were assigned to medication groups based on the bone-modifying agents initiated on the index date. We examined the association of bone-modifying agents and baseline parameters with hospitalization for any cause in this study population (the whole cohort). We also investigated the trajectory of serum calcium concentrations before and after the index date using a subset of patients excluding those without calcium measurements within the study window of 90 days before to 90 days after the index date (laboratory subset).

### Outcome assessment

Hospitalization for any cause was defined as the primary outcome in the whole cohort: admissions were identified from inpatient claims irrespective of the recoded diagnosis. For descriptive purposes, admissions were classified by the ICD-10 chapter of the diagnosis that consumed the most medical resources during the admission.

For the laboratory subset, the outcome was the longitudinal trajectory of albumin-corrected serum calcium levels from 90 days before to 90 days after the initiation of the medication. Corrected calcium levels were calculated using Payne’s equation [20]:


\begin{eqnarray*}
{\mathrm{Corrected}}\,{\mathrm{calcium}} &=& {\mathrm{measured}}\,{\mathrm{Ca}} + ( {4.0 - {\mathrm{albumin}}} ) \\&&\times \, 0.8\,{\mathrm{when}}\,{\mathrm{albumin}} \,< 4.0\,{\mathrm{g}}/{\mathrm{dl}}\\
\end{eqnarray*}

\begin{eqnarray*}
{\mathrm{Corrected}}\,{\mathrm{calcium}} = {\mathrm{measured}}\,{\mathrm{Ca}}\,{\mathrm{when}}\,{\mathrm{albumin}} \ge 4.0\,{\mathrm{g}}/{\mathrm{dl}}
\end{eqnarray*}

When albumin-adjusted calcium was directly reported in laboratory records, that value was used; otherwise it was calculated using the formula above.

The secondary outcome was hypocalcaemia, defined as a corrected serum calcium level below 8.0 mg/dl occurring within 90 days after medication initiation. The first occurrence was recorded in patients with multiple hypocalcaemic measurements. The threshold of 8.0 mg/dl was consistent with that used in pivotal randomized trials of bone-modifying agents [21–23]. In the sensitivity analysis, we applied a threshold of 8.4 mg/dl, defined as hypocalcaemia in the Japanese practical guidelines, and used in prior studies evaluating cardiovascular disease risk events [24, 25]. We also examined factors associated with hypercalcaemia defined as corrected serum calcium ≥10.1 mg/dl.

### Baseline covariates

Baseline characteristics were assessed within 30 days of the index date. Baseline demographics included age, sex, diabetes mellitus (DM; defined by diabetes medication use, HbA1c ≥6.5% [National Glycohemoglobin Standardization Program] or ≥6.1% [Japan Diabetes Society]), history of any recent hospitalization (within 1 year before index date), and history of cardiovascular disease (CVD), ascertained from all diagnosis codes recorded in the claims data using the code list in Supplementary Table S2. The medications included oral and intravenous vitamin D receptor activators (VDRA), calcimimetics, and phosphate-lowering agents (calcium- and iron-based) (Supplementary Table S1).

Laboratory data were measured locally and measurements were obtained from tests performed within 30 days before the index date: haemoglobin, total protein, albumin, creatinine, blood urea nitrogen, total cholesterol, triglycerides, low-density lipoprotein cholesterol, sodium, potassium, chloride, magnesium, calcium, phosphate, intact PTH, and C-reactive protein (CRP).

### Statistical analysis

The baseline characteristics are shown according to the type of bone-modifying agent used. Continuous variables are expressed as mean (standard deviation) or median (interquartile range), and categorical variables are expressed as numbers (%). Between-class comparisons were made using the Kruskal–Wallis test for nonparametric continuous variables, analysis of variance for parametric continuous variables, and chi-square test for categorical variables.

Factors associated with hospitalization for any cause were examined using a multivariable Cox proportional hazards model for the whole cohort. Laboratory data were available for only 374 patients (18.5%). We applied multiple imputation by chained equations (MICE) to the whole cohort with 100 imputations. Details are described in Supplementary Methods. The multivariable model included medication type (reference: BP), age, sex, DM, history of any recent hospitalization, history of CVD, VDRA use (oral or intravenous), calcium-based phosphate binder use, and calcimimetic use (Model 1). Model 2 further included baseline corrected calcium, phosphorus, log-transformed intact PTH, albumin, creatinine, log-transformed CRP, and haemoglobin. Further details for sensitivity analysis are provided in Supplementary Methods.

We examined the factors associated with hypocalcaemia and hypercalcaemia using multivariable logistic regression analysis. To address the missing laboratory data, we employed MICE with 50 imputations. The fully adjusted model included medication type (reference: BP), age, sex, DM, history of any recent hospitalization, history of CVD, VDRA use (oral or intravenous), calcium-based phosphate binder use, calcimimetic use, and baseline levels of corrected calcium, phosphorus, log-transformed intact PTH, and albumin. As a further exploratory analysis, restricted cubic splines were used to examine the nonlinear relationship between pretreatment levels of intact PTH and corrected serum calcium and the predicted probability of hypocalcaemia based on the multivariable logistic regression model.

All analyses were performed using StataNow MP version 19.5. Statistical significance was set at *P* < .05. No adjustment for multiple comparisons was made, because all analyses were exploratory.

## RESULTS

### Patient selection and baseline characteristics

We identified 41 167 patients who underwent dialysis for ≥90 days. Of these, 4322 were prescribed at least one bone-modifying agent. After excluding patients without a 6-month lookback period, 2020 patients were eligible for the primary analysis (whole cohort). Of these, 374 patients with serum calcium and albumin data available within 30 days of the index date comprised the laboratory subset (Fig. 1).

**Figure 1:**
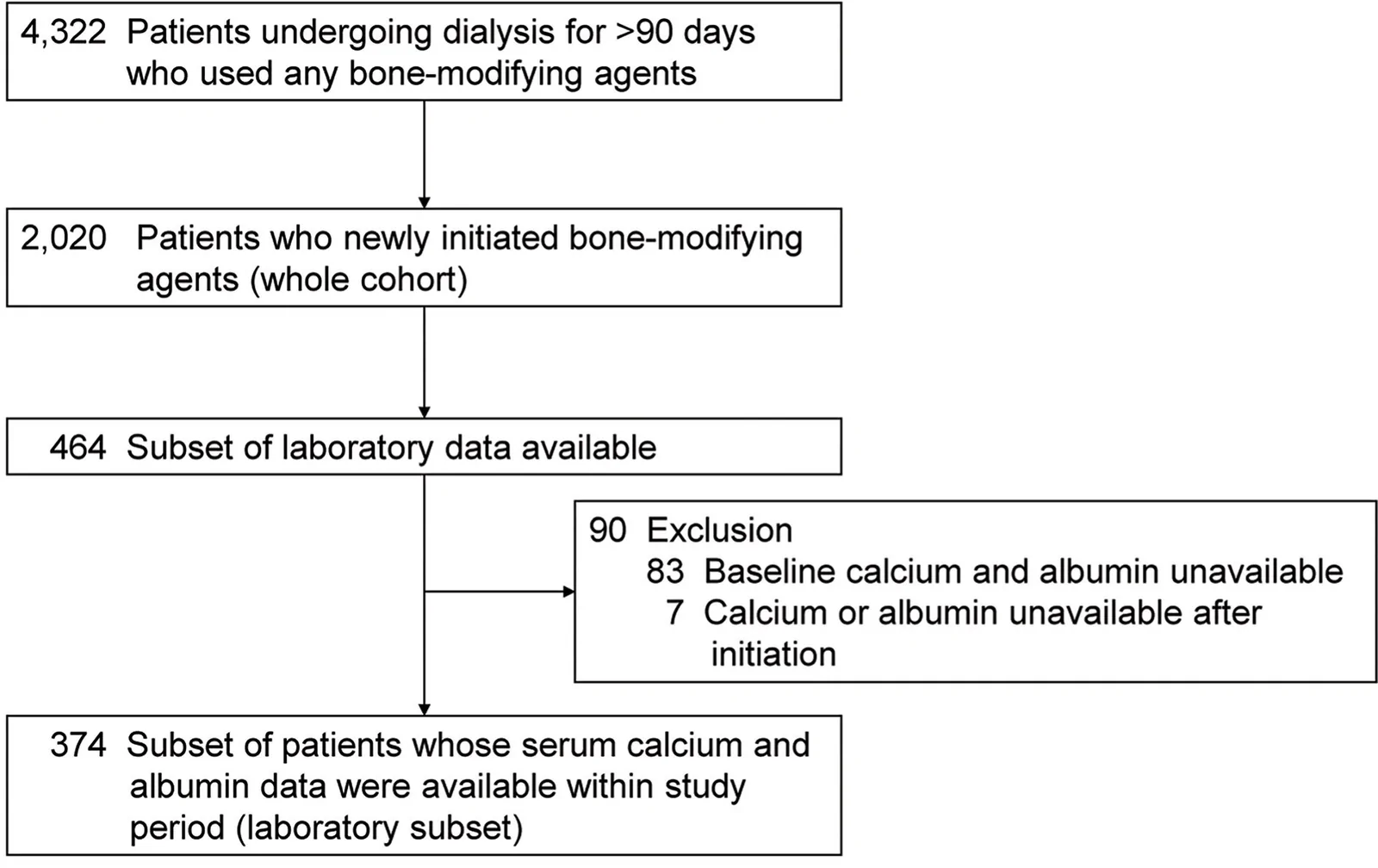
Patient selection flow. Flowchart showing the selection of the study population and analytic cohorts. The whole cohort included 2020 maintenance dialysis patients treated with bone-modifying agents, and 374 patients with available laboratory data comprised the laboratory subset.

The baseline characteristics of the whole cohort are shown in Table 1. The mean age was 73 years, and 45% of patients were men. Approximately 36% had DM, 54% had a history of hospitalization for any cause in the previous year, and 76% had a history of CVD. Fourteen patients (0.7%) underwent PD. Of the 2020 dialysis patients, 1057 (52.3%) received BP, 693 (34.3%) received DMAB, 136 (6.7%) received TPTD, and 134 (6.6%) received ROMO. Patients in the DMAB group were less likely to have a history of any recent hospitalization (48%) and more likely to be prescribed VDRA. Patients in the ROMO group were more likely to be prescribed calcium-based phosphate binders (39%) and less likely to be prescribed VDRA (57%). The history of any CVD was comparable across medication classes (*P* = .99). In the laboratory subset, corrected calcium levels varied across groups; the DMAB and ROMO groups were more likely to have higher calcium levels before initiation (Table 2).

**Table 1:** Baseline characteristics of the whole cohort.

	Total (*N* = 2020)	BP (*N* = 1057)	DMAB (*N* = 693)	TPTD (*N* = 136)	ROMO (*N* = 134)	*P* value
Age, years	73 (11)	72 (11)	72 (11)	75 (9)	74 (11)	.007
Male sex	901 (44.6)	473 (44.7)	311 (44.9)	64 (47.1)	53 (39.6)	.63
DM	721 (35.7)	389 (36.8)	244 (35.2)	51 (37.5)	37 (27.6)	.20
Modality						.006
HD	2006 (99.3)	1054 (99.7)	682 (98.4)	136 (100)	134 (100)	
PD	14 (0.7)	3 (0.3)	11 (1.6)	0 (0.0)	0 (0.0)	
History of any recent hospitalization	1098 (54.4)	584 (55.3)	335 (48.3)	102 (75.0)	77 (57.5)	<.001
History of any CVD	1530 (75.7)	802 (75.9)	522 (75.3)	104 (76.5)	102 (76.1)	.99
Heart failure	1377 (68.2)	717 (67.8)	474 (68.4)	90 (66.2)	96 (71.6)	.79
Stroke	424 (21.0)	227 (21.5)	144 (20.8)	32 (23.5)	21 (15.7)	.39
Coronary artery disease	384 (19.0)	211 (20.0)	117 (16.9)	32 (23.5)	24 (17.9)	.21
Calcimimetics	780 (38.6)	417 (39.5)	272 (39.2)	45 (33.1)	46 (34.3)	.36
VDRA	1428 (70.7)	738 (69.8)	534 (77.1)	80 (58.8)	76 (56.7)	<.001
PO	714 (35.3)	327 (30.9)	310 (44.7)	41 (30.1)	36 (26.9)	<.001
IV	799 (39.6)	445 (42.1)	264 (38.1)	45 (33.1)	45 (33.6)	.049
Phosphate binders	1422 (70.4)	752 (71.1)	492 (71.0)	88 (64.7)	90 (67.2)	.36
Calcium-based	627 (31.0)	320 (30.3)	217 (31.3)	37 (27.2)	53 (39.6)	.12
Iron-based	577 (28.6)	320 (30.3)	193 (27.8)	34 (25.0)	30 (22.4)	.17

Continuous values are expressed as mean (standard deviation), and categorical values are expressed as numbers (%).

BP, bisphosphonate; DMAB, denosumab; TPTD, teriparatide; ROMO, romosozumab; DM, diabetes mellitus; HD, haemodialysis; PD, peritoneal dialysis; CVD, cardiovascular disease; VDRA, vitamin D receptor agonist; PO, per oral; IV, intravenous.

**Table 2:** Baseline characteristics of the subset of laboratory parameters available.

	*N*	Total (*N* = 374)	BP (*N* = 237)	DMAB (*N* = 112)	TPTD (*N* = 10)	ROMO (*N* = 15)	*P* value
Age, years	374	72 (11)	73 (11)	72 (10)	80 (8)	68 (12)	.023
Male	374	180 (48.1)	119 (50.2)	53 (47.3)	3 (30.0)	5 (33.3)	.38
DM	374	161 (43.0)	99 (41.8)	52 (46.4)	6 (60.0)	4 (26.7)	.32
Modality	374						<.001
HD		366 (97.9)	237 (100)	104 (92.9)	10 (100)	15 (100)	
PD		8 (2.1)	0 (0.0)	8 (7.1)	0 (0.0)	0 (0.0)	
History of any recent hospitalization	374	210 (56.1)	136 (57.4)	56 (50.0)	9 (90.0)	9 (60.0)	.085
History of any CVD	374	303 (81.0)	186 (78.5)	95 (84.8)	8 (80.0)	14 (93.3)	.32
Heart failure	374	264 (70.6)	161 (67.9)	82 (73.2)	7 (70.0)	14 (93.3)	.18
Stroke	374	83 (22.2)	54 (22.8)	26 (23.2)	2 (20.0)	1 (6.7)	.52
Coronary artery disease	374	67 (17.9)	40 (16.9)	18 (16.1)	3 (30.0)	6 (40.0)	.094
Calcimimetics	374	154 (41.2)	95 (40.1)	46 (41.1)	2 (20.0)	11 (73.3)	.039
VDRA	374	271 (72.5)	171 (72.2)	86 (76.8)	5 (50.0)	9 (60.0)	.19
PO	374	158 (42.2)	90 (38.0)	59 (52.7)	5 (50.0)	4 (26.7)	.037
IV	374	128 (34.2)	90 (38.0)	32 (28.6)	1 (10.0)	5 (33.3)	.13
Phosphate binders	374	264 (70.6)	164 (69.2)	80 (71.4)	9 (90.0)	11 (73.3)	.55
Calcium-based	374	113 (30.2)	67 (28.3)	41 (36.6)	3 (30.0)	2 (13.3)	.20
Iron-based	374	128 (34.2)	92 (38.8)	29 (25.9)	2 (20.0)	5 (33.3)	.087
Laboratory data							
Haemoglobin, g/dl	374	11.1 (1.4)	11.1 (1.4)	11.1 (1.5)	11.3 (1.1)	11.6 (1.4)	.55
Total protein, g/dl	366	6.4 (0.7)	6.3 (0.6)	6.4 (0.6)	6.1 (1.0)	6.8 (0.9)	.036
Albumin, g/dl	374	3.4 (0.5)	3.4 (0.5)	3.5 (0.4)	3.4 (0.3)	3.5 (0.6)	.55
Creatinine, mg/dl	374	7.1 (3.2)	7.4 (3.2)	6.9 (3.2)	5.8 (2.8)	5.2 (2.4)	.020
BUN, mg/dl	374	44.7 (22.8)	46.0 (23.3)	42.7 (20.0)	47.3 (27.0)	37.1 (30.0)	.34
Total cholesterol, mg/dl	269	158.8 (37.4)	160.8 (38.2)	155.4 (33.6)	144.9 (43.3)	163.2 (47.4)	.49
TG, mg/dl	298	100.5 (51.2)	110.7 (57.3)	83.1 (34.3)	83.1 (27.3)	97.3 (43.2)	<.001
LDL cholesterol, mg/dl	238	82.4 (28.6)	83.7 (30.2)	81.1 (26.2)	77.3 (22.9)	54.0 (11.3)	.47
Sodium, mmol/l	367	138.1 (3.4)	138.2 (3.5)	138.0 (3.2)	137.5 (4.0)	137.1 (2.5)	.57
Potassium, mmol/l	374	4.2 (0.8)	4.2 (0.8)	4.1 (0.8)	4.1 (0.8)	4.0 (1.0)	.38
Chloride, mmol/l	374	103.1 (3.9)	103.1 (4.0)	103.3 (3.6)	103.3 (3.2)	102.4 (4.3)	.84
Magnesium, mg/dl	200	2.3 [2.1–2.5]	2.2 [2.0–2.5]	2.3 [2.1–2.5]	2.5 [2.4–2.6]	2.3 [2.2–3.0]	.17
Corrected calcium, mg/dl	374	9.2 (0.7)	9.2 (0.6)	9.5 (0.8)	9.1 (0.4)	9.3 (0.8)	.001
Phosphorus, mg/dl	372	4.2 (1.8)	4.1 (1.8)	4.4 (1.6)	3.3 (1.1)	3.2 (1.8)	.029
Intact PTH, pg/ml	241	125 [72–199]	135 [85–207]	122 [63–192]	109 [77–137]	72 [35–134]	.077
CRP, mg/dl	284	0.2 [0.1–0.6]	0.2 [0.1–0.6]	0.2 [0.0–0.6]	0.3 [0.1–1.7]	0.1 [0.1–0.2]	.62

Continuous variables are expressed as mean (standard deviation) or median (interquartile range), and categorical variables are expressed as numbers (%).

BP, bisphosphonate; DMAB, denosumab; TPTD, teriparatide; ROMO, romosozumab; DM, diabetes mellitus; HD, haemodialysis; PD, peritoneal dialysis; CVD, cardiovascular disease; VDRA, vitamin D receptor agonists; PO, per oral; IV, intravenous; BUN, blood urea nitrogen; TG, triglyceride; LDL, low-density lipoprotein; PTH, parathyroid hormone; CRP, C-reactive protein.

### Post-treatment hospitalization and the associated factors

Among 2020 patients, 425 were hospitalised for any cause within 90 days of treatment initiation (Supplementary Table S3). Circulatory causes, injury and poisoning, and genitourinary causes accounted for 17.7%, 20.7%, and 24.9%, respectively. The cumulative incidence of hospitalization did not differ across medication classes (Fig. 2). A history of any recent hospitalization and lower corrected serum calcium levels were independently associated with post-treatment hospitalization [adjusted hazard ratios (HR) and 95% confidence intervals (CI) were 2.18 (1.64, 2.90) and 0.69 (0.50, 0.97) per 1 mg/dl increase, respectively], and medication class was not associated with hospitalization events (Table 3). The sensitivity analysis results were consistent with those of the main analysis, although not statistically significant in the complete-case analysis (Supplementary Table S4).

**Figure 2:**
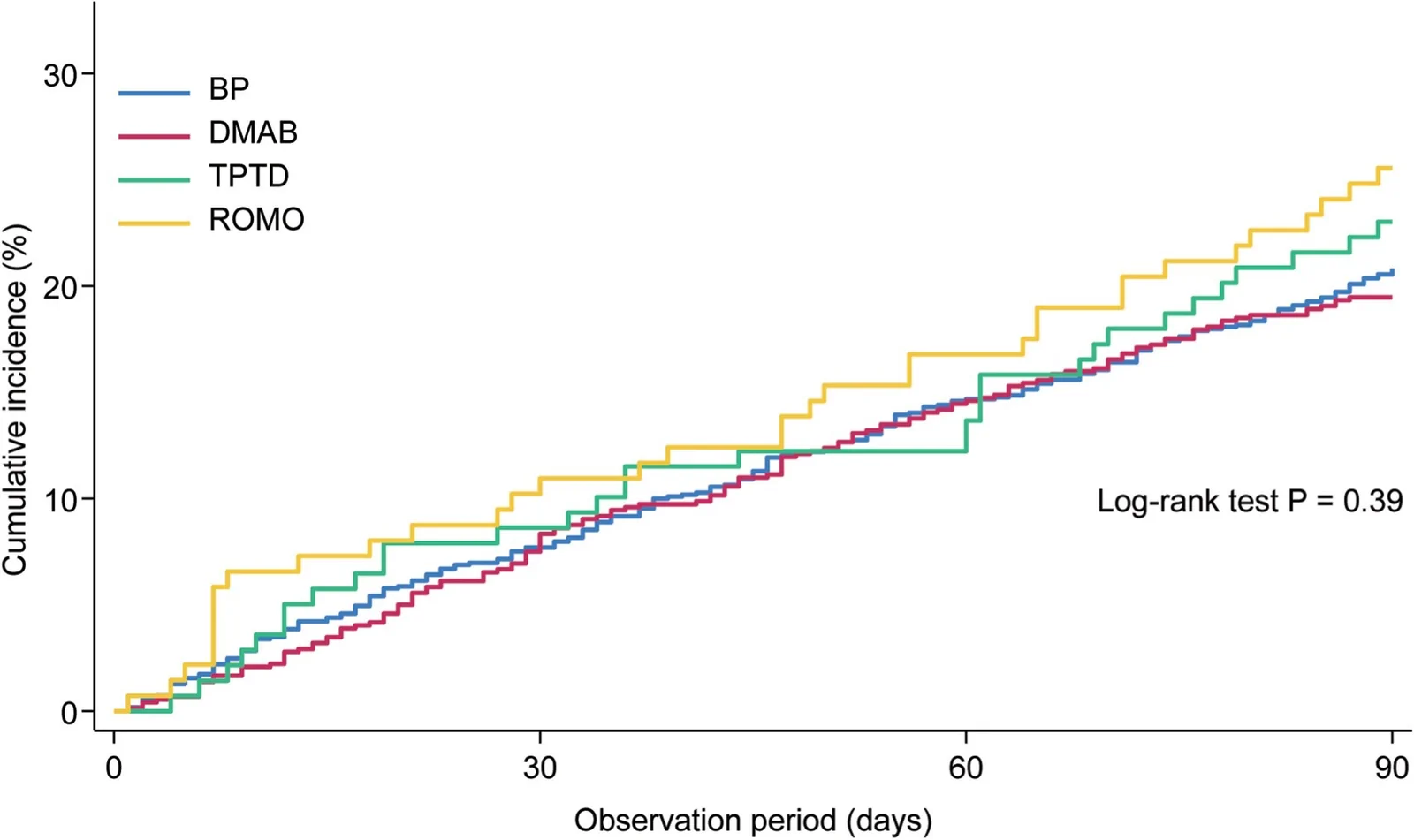
Cumulative incidence of all-cause hospitalization within 90 days of the initiation of bone-modifying agents. The cumulative incidence of all-cause hospitalization within 90 days of the initiation of bone-modifying agents was estimated using the Kaplan-Meier method. No significant differences were observed across medication classes (log-rank *P* = .29). BP, bisphosphonate; DMAB, denosumab; ROMO, romosozumab; TPTD, teriparatide.

**Table 3:** Factors associated with all-cause hospitalization in the whole cohort (*n* = 2020).

	Model 1		Model 2	
Parameters	HR (95% CI)	*P* values	HR (95% CI)	*P* values
BP	Reference	–	Reference	–
DMAB	0.97 (0.78, 1.20)	.79	1.06 (0.81, 1.38)	.69
TPTD	0.94 (0.65, 1.37)	.75	0.94 (0.56, 1.56)	.80
ROMO	1.21 (0.85, 1.74)	.29	1.32 (0.79, 2.20)	.29
Age	1.01 (1.00, 1.02)	.13	1.00 (0.99, 1.01)	.81
Male sex	0.98 (0.81, 1.18)	.81	0.91 (0.71, 1.17)	.48
DM	1.02 (0.83, 1.24)	.86	0.99 (0.78, 1.24)	.92
History of any recent hospitalization	2.26 (1.81, 2.82)	<.001	2.18 (1.64, 2.90)	<.001
History of CVD	1.22 (0.96, 1.56)	.11	1.26 (0.95, 1.66)	.11
VDRA	0.88 (0.72, 1.09)	.24	0.90 (0.70, 1.15)	.40
Calcium-based phosphate binders	1.06 (0.86, 1.31)	.60	1.08 (0.84, 1.38)	.56
Calcimimetics	1.06 (0.86, 1.29)	.60	1.05 (0.83, 1.32)	.71
Corrected calcium			0.69 (0.50, 0.97)	.030
Phosphorus			1.07 (0.93, 1.23)	.33
Log-transformed intact PTH			0.92 (0.68, 1.23)	.57
Albumin			0.81 (0.45, 1.44)	.46
Creatinine			0.99 (0.93, 1.06)	.83
Log-transformed CRP			1.07 (0.91, 1.26)	.41
Haemoglobin			1.01 (0.86, 1.18)	.92

HR, hazard ratio; CI, confidence interval; BP, bisphosphonate; DMAB, denosumab; TPTD, teriparatide; ROMO, romosozumab; DM, diabetes mellitus; HD, haemodialysis; PD, peritoneal dialysis; CVD, cardiovascular disease; VDRA, vitamin D receptor agonists; PO, per oral; IV, intravenous; PTH, parathyroid hormone; CRP, C-reactive protein.

### Serum calcium trajectory, post-treatment hypocalcaemia, and its associated factors

Among the 374 patients with laboratory data, we examined the trajectory of corrected serum calcium levels (Fig. 3). Serum calcium levels remained persistently low after DMAB or ROMO initiation, whereas in patients who initiated BP and TPTD, serum calcium levels remained stable throughout the observation period.

**Figure 3:**
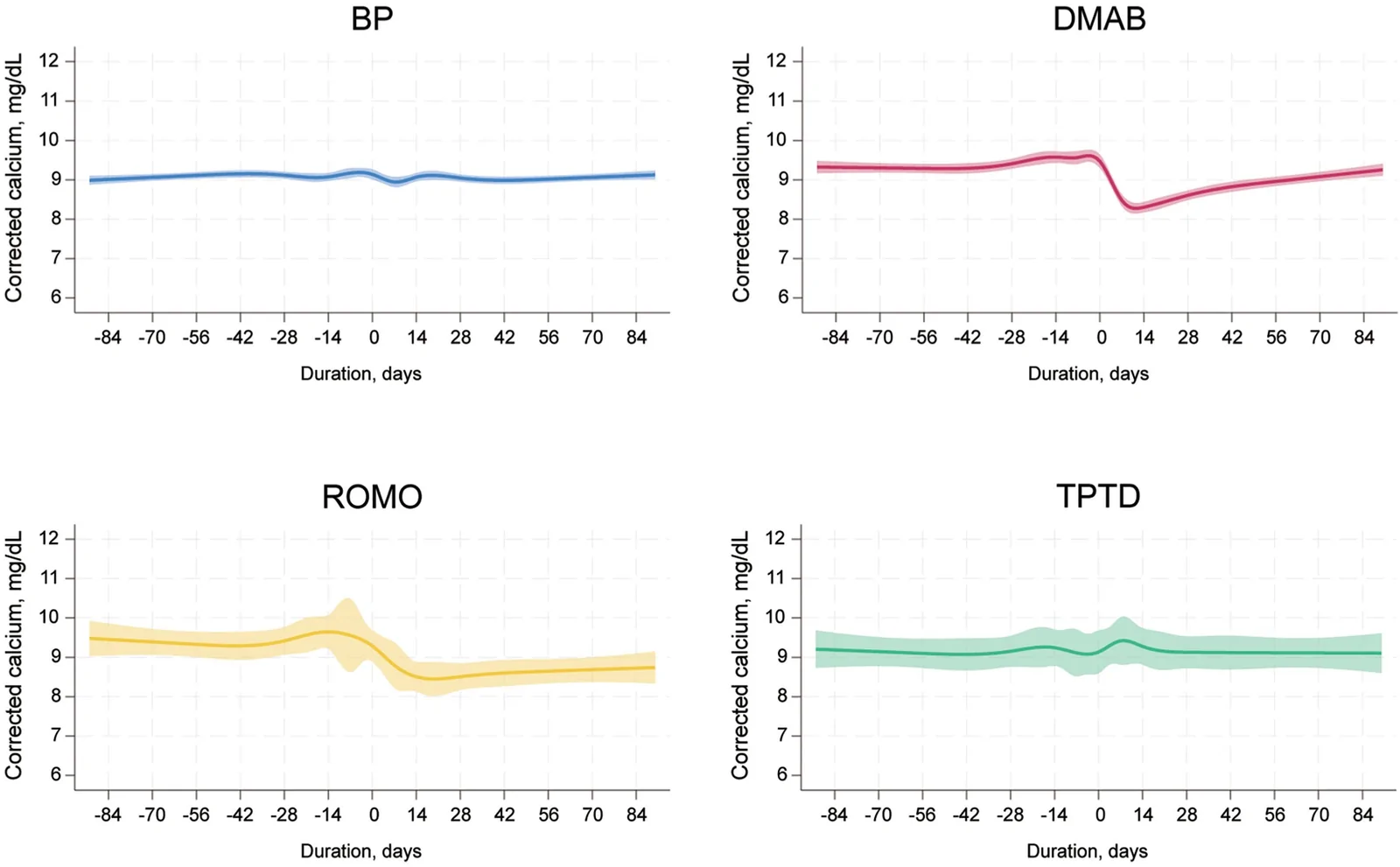
Corrected serum calcium trajectories within 90 days of the initiation of bone-modifying agents. Trajectories of corrected serum calcium levels from 90 days before to 90 days after the initiation of bone-modifying agent treatment were estimated using restricted cubic splines with mixed-effects models. The shaded areas represent the 95% confidence intervals. BP, bisphosphonate; DMAB, denosumab; ROMO, romosozumab; TPTD, teriparatide.

Hypocalcaemia (<8.0 mg/dl) occurred in 99 (26.5%) patients; 35 of 237 BP users (14.8%); 57 of 112 DMAB users (50.9%); 0 of 10 TPTD users (0%); and 7 of 15 ROMO users (46.7%). Owing to the absence of hypocalcaemia events among TPTD users, the association between TPTD and hypocalcaemia could not be estimated; therefore, multivariable analysis was restricted to the remaining 364 patients after excluding TPTD users. Compared with BP, DMAB and ROMO were significantly associated with hypocalcaemia with adjusted odds ratios (OR) and 95% CI of 13.4 (6.90, 26.1) and 8.09 (2.20, 29.8), respectively. Pretreatment calcium and intact PTH levels were associated with post-treatment hypocalcaemia with adjusted OR and CI of 0.29 (0.18, 0.49) and 1.59 (1.05, 2.39), respectively (Table 4). In the sensitivity analysis of different definitions of hypocalcaemia (<8.4 mg/dl), the results were largely consistent, except for VDRA use, with an OR and CI of 0.50 (0.28, 0.90).

**Table 4:** Factors associated with hypocalcaemia in dialysis patients who initiated osteoporosis medications (*n* = 364).

Observation: 364^a^	cCa < 8.0 mg/dl		cCa < 8.4 mg/dl	
Parameters	OR (95% CI)	*P* values	OR (95% CI)	*P* values
BP	Reference		Reference	
DMAB	13.4 (6.90, 26.1)	<.001	10.2 (5.29, 19.7)	<.001
ROMO	8.09 (2.20, 29.8)	.002	4.66 (1.21, 17.9)	.025
Age	0.98 (0.95, 1.01)	.13	0.98 (0.96, 1.01)	.15
Male sex	0.86 (0.49, 1.51)	.59	0.89 (0.53, 1.48)	.64
DM	0.97 (0.54, 1.74)	.91	1.42 (0.82, 2.44)	.21
History of any recent hospitalization	1.27 (0.69, 2.32)	.45	0.72 (0.42, 1.25)	.25
History of CVD	0.64 (0.31, 1.33)	.23	0.87 (0.45, 1.70)	.69
VDRA	0.58 (0.31, 1.07)	.082	0.50 (0.28, 0.90)	.022
Calcium-based phosphate binders	0.89 (0.48, 1.64)	.70	1.01 (0.57, 1.77)	.98
Calcimimetics	1.10 (0.61, 1.98)	.74	1.33 (0.78, 2.28)	.30
Corrected calcium	0.29 (0.18, 0.49)	<.001	0.22 (0.14, 0.36)	<.001
Phosphorus	0.88 (0.75, 1.04)	.13	1.02 (0.88, 1.18)	.80
Log-transformed intact PTH	1.59 (1.05, 2.39)	.027	1.69 (1.09, 2.62)	.019
Albumin	1.21 (0.62, 2.35)	.57	1.22 (0.67, 2.22)	.53

^a^Due to the absence of hypocalcaemia events in the TPTD group, 10 patients (TPTD users) were excluded.

cCa, corrected serum calcium; OR, odds ratio; CI, confidence interval; BP, bisphosphonate; DMAB, denosumab; ROMO, romosozumab; DM, diabetes mellitus; CVD, cardiovascular disease; VDRA, vitamin D receptor agonists; PTH, parathyroid hormone; TPTD, teriparatide.

In exploratory analyses using restricted cubic splines, the predicted probability of hypocalcaemia increased monotonically with higher pretreatment intact PTH levels without a clear threshold effect, whereas a markedly elevated risk was observed in patients with pretreatment corrected serum calcium <9.0 mg/dl (Fig. 4).

**Figure 4:**
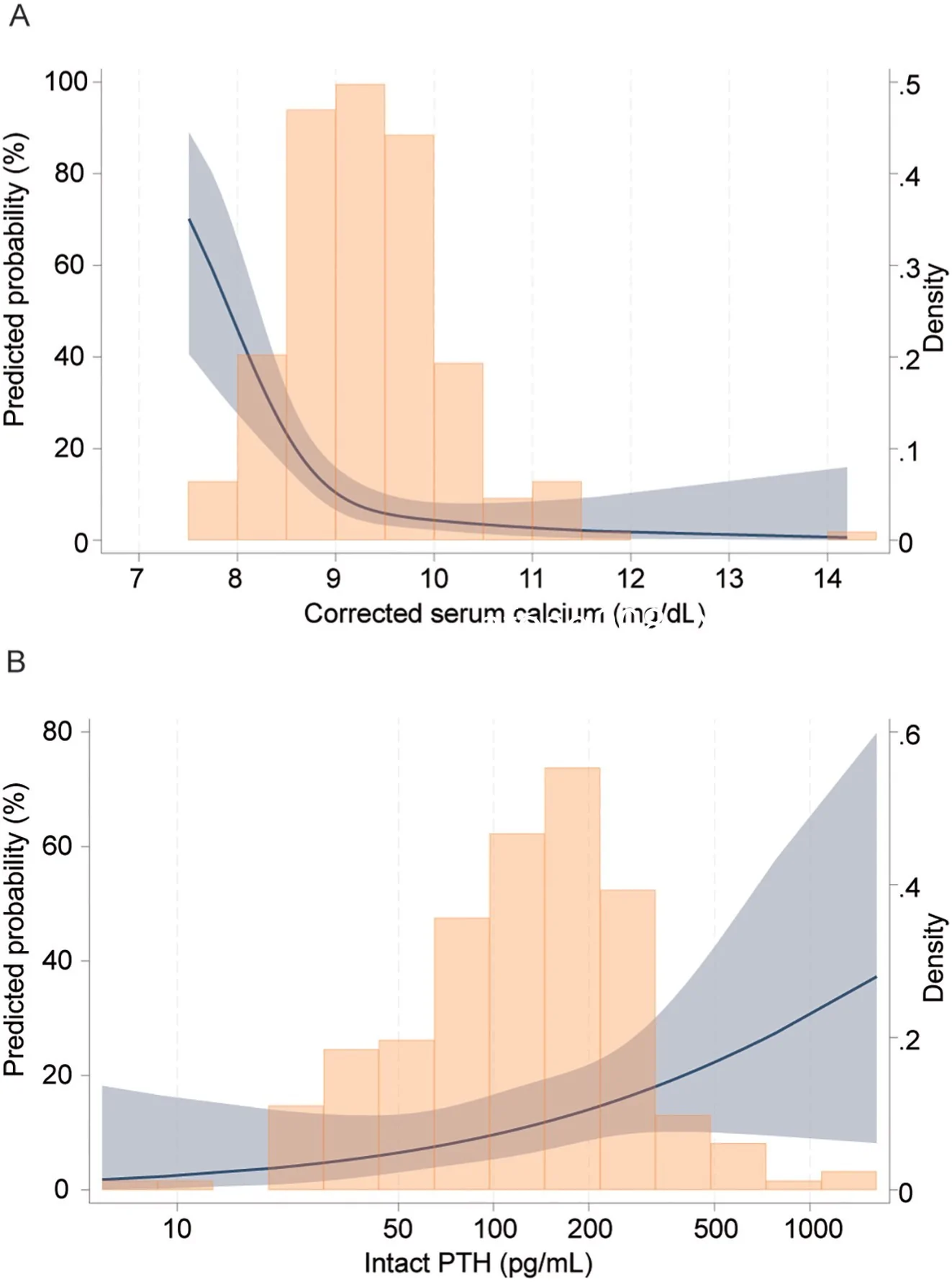
Predicted probability of hypocalcaemia by pretreatment corrected serum calcium and intact PTH levels. Predicted probability of hypocalcaemia estimated from multivariable logistic regression models with restricted cubic splines according to pretreatment corrected serum calcium levels (A) and pretreatment intact PTH levels (B). The predicted probability of hypocalcaemia increased monotonically with higher pretreatment intact PTH levels without an identifiable threshold, whereas the post-treatment hypocalcaemia risk was markedly elevated in patients with pretreatment corrected serum calcium levels below 9.0 mg/dl.

Hypercalcaemia (≥10.1 mg/dl) occurred in 69 (18.4%) patients, with no significant differences across medication classes. Calcium-based phosphate binder use and higher pretreatment corrected serum calcium were associated with post-treatment hypercalcaemia (Supplementary Table S5).

## DISCUSSION

In this real-world cohort of maintenance dialysis patients receiving bone-modifying agents, we made three key observations. First, lower corrected serum calcium levels before initiating bone-modifying agents were independently associated with hospitalization for any cause, suggesting that pretreatment hypocalcaemia may have clinical consequences extending beyond laboratory abnormality. Second, DMAB and ROMO were associated with markedly higher rates of hypocalcaemia than BP, with persistently low serum calcium levels after initiation. Third, the predicted probability of hypocalcaemia increased with elevated pretreatment intact PTH levels and decreased with pretreatment corrected serum calcium levels.

The association between lower pretreatment corrected serum calcium levels and all-cause hospitalization is consistent with prior evidence linking hypocalcaemia to adverse cardiovascular outcomes in dialysis patients [25, 26]. Hypocalcaemia may precipitate a range of clinical events—including neuromuscular symptoms, arrhythmias, and cardiac dysfunction—that collectively increase hospitalization risk [27]. The heterogeneous composition of hospitalizations indicates that the association with lower pretreatment calcium is unlikely to reflect cardiovascular events alone. Genitourinary diagnoses, the most frequent category, probably reflect the coding of kidney replacement therapy itself rather than an acute illness, whereas injury and poisoning diagnoses may partly reflect fractures. These patterns illustrate the difficulty of interpreting ICD-10-coded causes in administrative databases. All-cause hospitalization was selected as the primary outcome in part because heart failure cannot be reliably distinguished from volume overload in patients receiving maintenance dialysis; among randomized trials in this population that reported heart failure events, none specified criteria separating the two entities [28]. No significant difference in hospitalization was observed across medication classes; however, this null finding should be interpreted with caution, as the study was not powered to detect such differences. Nonetheless, pretreatment hypocalcaemia was independently associated with hospitalization, regardless of medication type.

DMAB and ROMO were associated with markedly higher odds of post-treatment hypocalcaemia than BP. A study reported a 12-week cumulative incidence of severe hypocalcaemia of 41% with DMAB versus 2% with oral BP in female dialysis-dependent patients, leading to a warning for DMAB in patients with advanced CKD in 2024 [14]. The pronounced risk of hypocalcaemia in patients on dialysis reflects both drug-specific mechanisms and population-specific vulnerability. DMAB abruptly suppresses osteoclast activity via RANKL inhibition, eliminating bone resorption as a calcium source, and inducing a ‘hungry bone’-like state, particularly in patients with high-turnover bone disease due to secondary hyperparathyroidism [29]. ROMO produces a comparable effect through a dual mechanism: sclerostin inhibition simultaneously promotes bone formation and suppresses resorption, further increasing the demand for skeletal calcium. These effects are amplified in patients undergoing dialysis, who lack the capacity to upregulate renal tubular calcium reabsorption or synthesize active vitamin D in response to hypocalcaemia. The persistently low serum calcium levels observed following initiation in our study are consistent with prolonged hungry bone physiology. Notably, pivotal trials of both DMAB and ROMO systematically excluded patients on dialysis, underscoring the real-world value of our findings [21–23].

The observed comparisons are shaped not only by pharmacology but also by prescribing patterns. Although BP served as the reference category and did not demonstrate a statistically significant association in relative terms, the observed incidence of hypocalcaemia in BP users (14.8%) was not negligible. This is consistent with a previous study in Japan that reported an increased risk of hypocalcaemia with BP in patients with decreased kidney function [13]. The attenuated relative risk compared with DMAB and ROMO likely reflects pharmacological differences rather than the absence of risk, as BP suppresses bone resorption less potently and more gradually. Therefore, BP should be regarded as comparatively safer, but not free of risk. Conversely, the estimates for anabolic agents may be attenuated by patient selection. Patients initiating TPTD and ROMO were older, had lower intact PTH levels, and received less intensive therapy for secondary hyperparathyroidism—a profile suggestive of low bone turnover. This suggests that anabolic agents may be preferentially selected for patients with low-turnover bone, and residual confounding by bone turnover status cannot be excluded. In terms of cardiovascular risk, however, romosozumab users were not a lower-risk group: the prevalence of prior cardiovascular disease was comparable across medication classes. Given the cardiovascular safety signal observed for romosozumab in trials, this suggests that in real-world practice it was not preferentially avoided in patients with established cardiovascular disease.

Elevated pretreatment PTH levels were independently associated with post-treatment hypocalcaemia across medication classes, consistent with the concept that high bone turnover predisposes patients to a hungry bone state upon abrupt suppression of bone resorption [14, 29]. In patients with advanced CKD, PTH serves as a useful population-level surrogate for bone turnover, with markedly elevated levels indicating high-turnover bone pathology [8]. Experimental evidence has shown that anti-sclerostin antibody therapy increases bone when secondary hyperparathyroidism is suppressed but remains attenuated in high-PTH states [30], implying that in such settings, the osteoanabolic efficacy of ROMO may be blunted, yet hypocalcaemia still ensues. Pretreatment corrected serum calcium below 9.0 mg/dl was associated with a markedly elevated probability of post-treatment hypocalcaemia, and optimising pretreatment calcium may confer the additional benefit of suppressing PTH. In exploratory analyses of hypercalcaemia, calcium-based phosphate binder use, but not bone-modifying agent class, was associated with post-treatment hypercalcaemia, suggesting that active vitamin D analogues may be preferable when correcting pretreatment hypocalcaemia—consistent with current guidelines recommending restriction of calcium-based phosphate binders. These findings underscore that pretreatment evaluation of PTH and calcium homeostasis is critical, regardless of the intended medication.

This study had some limitations. First, as an observational study, residual confounding cannot be excluded, including confounding by indication, whereby clinicians may preferentially prescribe BP to patients who are perceived to be at a higher risk of hypocalcaemia. Second, the JMDC hospital data lacked bone mineral density measurements and direct bone turnover markers, such as P1NP, TRACP-5b, and CTX. Standardized ALP was also unavailable due to changes in the ALP assay throughout the study period. Instead, we used intact PTH as a surrogate for bone turnover, which has recognised limitations in this population. Third, laboratory data were available for only 374 patients (18.5%), and although multiple imputations were applied under the missing-at-random assumption, this assumption could not be formally verified. Fourth, the small number of ROMO and TPTD users resulted in wide confidence intervals or inestimable associations with the outcome, limiting the precision of the estimates for these agents. Therefore, the results should be regarded as exploratory. Fifth, the proportion of patients undergoing peritoneal dialysis was quite low, which may limit generalizability to the peritoneal dialysis population, in whom calcium balance differs owing to dialysate composition and continuous clearance. Finally, albumin-corrected serum calcium correlates poorly with ionised calcium in dialysis patients, particularly in the hypocalcaemic range, tending to overestimate calcium status [31, 32]. This may have underestimated the true incidence of hypocalcaemia, biasing associations towards the null.

In this real-world cohort of maintenance dialysis patients, DMAB and ROMO were associated with a markedly higher and more prolonged hypocalcaemia risk than BP, and a lower corrected serum calcium level was independently associated with all-cause hospitalization. Elevated pretreatment PTH levels emerged as a key predictor of post-treatment hypocalcaemia across medication classes. These findings underscore the importance of optimising CKD-MBD management, particularly PTH control and calcium homeostasis before initiating treatment with bone-modifying agents in patients undergoing dialysis.

## Supplementary Material

sfag364_Supplemental_File

## Data Availability

The data used in this study were obtained from the JMDC database, a third-party source. Because of licensing agreements with JMDC Inc., the data are not publicly available to other researchers. Information on the original dataset is available on the JMDC website (https://www.jmdc.co.jp/en/jmdc-claims-database/).
