# Supplementary material for: Hypocalcaemia and hospitalization after initiation of bone-modifying agents in dialysis patients
Source: Clin Kidney J. 2026 Nov 2;19(11):sfag364. doi: 10.1093/ckj/sfag364 (PMC13760220; doi:10.1093/ckj/sfag364)
Supplement: Supplementary file 1 [file sfag364_supplemental_file.docx]

**Hypocalcaemia and All-Cause Hospitalisation Following Bone-Modifying Agent Initiation in Maintenance Dialysis Patients: A Real-World Cohort Study**

Authors: Takahiro Imaizumi^1,2*^, Nobuhiro Nishibori^1^, Masaki Okazaki^1,2,4^, Koya Nagase^1^, Yuko Asano^1,2^, Yoshihisa Takada^2^, Tomoko Handa^2^, Daisuke Ito^2^, Atsushi Hashizume^2,3^, Shoichi Maruyama^1^

**Supplementary Material**

[Supplementary Methods	2](#_Toc238323124)

[Supplementary Table 1. Medications included in the study, classified by ATC code	4](#_Toc238323125)

[Supplementary Table 2. List of disease codes defining cardiovascular disease	5](#_Toc238323126)

[Supplementary Table 3. Distribution of the diagnosis consuming the most medical resources at admission, classified by ICD-10 chapter	6](#_Toc238323128)

[Supplementary Table 4. Factors associated with any hospitalization in the laboratory subset (n = 374) and the complete-case subset (n = 243)	7](#_Toc238323130)

[Supplementary Table 5. Factors associated with hypercalcaemia (n = 364)	8](#_Toc238323131)

Supplementary Methods

*Details in statistical analysis (multiple imputations)*

For the analysis of Cox proportional hazards model examining factors associated with hospitalisation for any cause, we applied multiple imputations by chained equations to the whole cohort. Given the high fraction of missing information (the fraction of missing information [FMI] = 0.86) observed for laboratory variables, we used 100 imputation datasets, consistent with the recommendation that the number of imputations should approximate the largest FMI expressed as a percentage*. The imputation model included all covariates in the analytical model, with PTH and CRP levels log-transformed before imputation to normalize their distributions.

For the hypocalcaemia analysis, which was restricted to the laboratory subset, the fraction of missing information was substantially lower (FMI = 0.43); accordingly, 50 imputations were used.

*von Hippel PT (2020) How Many Imputations Do You Need? A Two-stage Calculation Using a Quadratic Rule. Sociol Methods Res 49:699–718.

*Sensitivity analysis*

For sensitivity analysis, we repeated the analysis in the laboratory subset (n=374, with multiple imputation for residual missingness) and in a complete-case subset restricted to patients with all covariates available (n = 243), with adjustment for variables in Model 1 and baseline corrected calcium, phosphorus, log-transformed intact PTH because of limited sample size.

*Mixed-effects models and trajectory of corrected serum calcium concentration*

Mixed-effects models were used to describe the longitudinal trajectory of corrected calcium levels, with patient-level random intercepts to account for repeated measurements within individuals. Time was modelled using restricted cubic splines with knots at -56, -28, -14, -7, 0, 7, 14, 28, and 56 days relative to the index date to capture non-linear temporal patterns. We tested the interaction between medication type and time splines to evaluate whether calcium trajectories differed according to medication class. The model specifications are as follows.

cCa_it = β₀ + β₁×Type + β₂×f(Time) + β₃×Type×f(Time) + u_i + ε_it

where f(Time) represents the cubic spline function, Type indicates medication category, u_i is the patient-specific random intercept, and ε_it is the residual error.

Supplementary Table 1. Medications included in the study, classified by ATC code

| Category | ATC code | Generic name |
| --- | --- | --- |
| Bone-modifying agents |  |  |
| BP | M05BA02 | *Etidronate* |
| BP | M05BA04 | *Alendronate* |
| BP | M05BA05 | *Minodronate* |
| BP | M05BA06 | *Ibandronate* |
| BP | M05BA07 | *Risedronate* |
| BP | M05BA08 | *Zoledronate* |
| DMAB | M05BX04 | *Denosumab* |
| ROMO | M05BX06 | *Romosozumab* |
| TPTD | H05AA02 | *Teriparatide (recombinant)* |
| TPTD | H05AA02 | *Teriparatide acetate* |
| Active vitamin D |  |  |
| VitD | A11CC03 | *Alfacalcidol* |
| VitD | A11CC04 | *Calcitriol* |
| VitD | A11CC05 | *Falecalcitriol* |
| VitD | A11CC06 | *Eldecalcitol* |
| VitD | A11CC10 | *Maxacalcitol* |
| Phosphate-lowering agents |  |  |
| Calcium-based binders | A12AA04 | *Calcium carbonate* |
| Non-calcium-based binders | V03AE02 | *Sevelamer carbonate* |
| Non-calcium-based binders | V03AE03 | *Lanthanum carbonate* |
| Non-calcium-based binders | V03AE04 | *Sucroferric oxyhydroxide* |
| Non-calcium-based binders | V03AE05 | *Ferric citrate* |
| Non-calcium-based binders | V03AE07 | *Bixalomer* |
| Intestinal phosphate absorption inhibitor | A06AX11 | *Tenapanor* |
| Calcimimetics | H05BX01 | *Cinacalcet* |
| Calcimimetics | H05BX04 | *Etelcalcetide* |
| Calcimimetics | H05BX05 | *Evocalcet* |
| Calcimimetics | H05BX06 | *Upacicalcet* |

ATC = Anatomical Therapeutic Chemical classification (WHO). BP = bisphosphonate; DMAB = denosumab; ROMO = romosozumab; TPTD = teriparatide; PTHa = PTH analogue; Paricalcitol and doxercalciferol (used in other countries for secondary hyperparathyroidism) are classified under H05BX and are not included as they are not approved in Japan.

Supplementary Table 2. List of disease codes defining cardiovascular disease

| **Disease category** | **ICD-10 codes** |
| --- | --- |
| Heart failure | I09.9, I11.0, I13.0, I13.2, I25.5, I42.0, I42.5-I42.9, I43.x, I50.x |
| Stroke | G45.x, G46.x, I60.x, I61.x, I63.x, I64, H34.1 |
| Coronary artery disease | I21.x, I22.x, I23.x |

ICD-10, International Statistical Classification of Diseases and Related Health Problems, 10th Revision. Definitions are based on the article*.

*Goto H, Iseri K, Hida N. Fibrates and the risk of cardiovascular outcomes in chronic kidney disease patients. Nephrol Dial Transplant. 2024; 39(6): 1016-1022. doi: 10.1093/ndt/gfad248. PMID: 38012115; PMCID: PMC11139516.

**Supplementary Table 3. Distribution of the diagnosis consuming the most medical resources at admission, classified by ICD-10 chapter**

| No | Chapter | ICD-10 | Whole cohort | Laboratory subset |
| --- | --- | --- | --- | --- |
| I | Certain infectious and parasitic diseases | A00-B99 | 16 (3.8) | 0 |
| II | Neoplasms | C00-D48 | 19 (4.5) | 5 (5.8) |
| III | Diseases of the blood and blood-forming organs and certain disorders involving the immune mechanism | D50-D89 | 4 (0.9) | 1 (1.2) |
| IV | Endocrine, nutritional and metabolic diseases | E00-E90 | 9 (2.1) | 2 (2.3) |
| V | Mental and behavioural disorders | F00-F99 | 0 | 0 |
| VI | Diseases of the nervous system | G00-G99 | 5 (1.2) | 0 |
| VII | Diseases of the eye and adnexa | H00-H59 | 7 (1.7) | 3 (3.5) |
| VIII | Diseases of the ear and mastoid process | H60-H95 | 2 (0.5) | 1 (1.2) |
| IX | Diseases of the circulatory system | I00-I99 | 75 (17.7) | 18 (20.7) |
| X | Diseases of the respiratory system | J00-J99 | 25 (5.9) | 7 (8.1) |
| XI | Diseases of the digestive system | K00-K93 | 37 (8.7) | 3 (3.5) |
| XII | Diseases of the skin and subcutaneous tissue | L00-L99 | 5 (1.2) | 1 (1.2) |
| XIII | Diseases of the musculoskeletal system and connective tissue | M00-M99 | 15 (3.5) | 1 (1.2) |
| XIV | Diseases of the genitourinary system | N00-N99 | 106 (24.9) | 20 (23.0) |
| XV | Pregnancy, childbirth and the puerperium | O00-O99 | 0 | 0 |
| XVI | Certain conditions originating in the perinatal period | P00-P96 | 0 | 0 |
| XVII | Congenital malformations, deformations and chromosomal abnormalities | Q00-Q99 | 2 (0.5) | 0 |
| XVIII | Symptoms, signs and abnormal clinical and laboratory findings, not elsewhere classified | R00-R99 | 0 | 0 |
| XIX | Injury, poisoning and certain other consequences of external causes | S00-T98 | 88 (20.7) | 24 (27.6) |
| XX | External causes of morbidity and mortality | V01-Y98 | 0 | 0 |
| XXI | Factors influencing health status and contact with health services | Z00-Z99 | 0 | 0 |
| XXII | Codes for special purposes | U00-U85 | 10 (2.4) | 1 (1.2) |

ICD-10, International Statistical Classification of Diseases and Related Health Problems, 10th Revision. The diagnosis consuming the most medical resources is recorded. Diagnoses were coded using the Japanese version of ICD-10 (2013 revision)

**Supplementary Table 4. Factors associated with any hospitalization in the laboratory subset (n** = 374) and the complete-case subset (n = 243)

|  | Laboratory subset | | Complete-case subset | |
| --- | --- | --- | --- | --- |
| Parameters | HR (95% CI) | P values | HR (95% CI) | P values |
| BP | Reference | – | Reference | – |
| DMAB | 0.85 (0.51, 1.43) | 0.55 | 1.02 (0.57, 1.82) | 0.94 |
| TPTD | 0.61 (0.14, 2.57) | 0.50 | 1.01 (0.23, 4.38) | 0.99 |
| ROMO | 0.90 (0.32, 2.58) | 0.85 | 0.89 (0.30, 2.64) | 0.84 |
| Age | 1.00 (0.98, 1.02) | 0.69 | 1.00 (0.97, 1.02) | 0.74 |
| Male sex | 1.08 (0.70, 1.65) | 0.74 | 1.03 (0.63, 1.68) | 0.92 |
| DM | 0.85 (0.54, 1.33) | 0.47 | 0.79 (0.47, 1.33) | 0.37 |
| History of any recent hospitalization | 2.31 (1.42, 3.78) | 0.001 | 2.03 (1.14, 3.61) | 0.016 |
| History of CVD | 1.77 (0.93, 3.38) | 0.083 | 2.46 (0.98, 6.21) | 0.056 |
| VDRA | 0.76 (0.47, 1.22) | 0.25 | 0.75 (0.43, 1.31) | 0.31 |
| Calcium-based phosphate binders | 0.76 (0.46, 1.26) | 0.30 | 0.62 (0.35, 1.12) | 0.11 |
| Calcimimetics | 1.53 (0.98, 2.38) | 0.063 | 1.23 (0.73, 2.07) | 0.44 |
| Corrected Calcium | 0.68 (0.47, 0.98) | 0.037 | 0.76 (0.49, 1.16) | 0.20 |
| Phosphorus | 1.05 (0.93, 1.18) | 0.46 | 1.07 (0.93, 1.23) | 0.35 |
| Log-transformed intact PTH | 0.99 (0.74, 1.32) | 0.92 | 0.97 (0.71, 1.32) | 0.84 |

HR, hazard ratio; CI, confidence interval; BP, bisphosphonate; DMAB, denosumab; TPTD, teriparatide; ROMO, romosozumab; DM, diabetes mellitus; CVD, cardiovascular disease; VDRA, vitamin D receptor agonists; PTH, parathyroid hormone.

**Supplementary Table 5. Factors associated with hypercalcaemia (n** = 364)

| Parameters (n = 364*) | **OR (95% CI)** | **P values** |
| --- | --- | --- |
| BP | Reference | – |
| DMAB | 0.82 (0.42, 1.61) | 0.57 |
| ROMO | 0.98 (0.21, 4.55) | 0.98 |
| Age | 1.00 (0.97, 1.03) | 0.95 |
| Male sex | 0.48 (0.25, 0.91) | 0.024 |
| DM | 1.09 (0.57, 2.07) | 0.80 |
| History of any recent hospitalization | 1.73 (0.89, 3.38) | 0.11 |
| History of CVD | 1.01 (0.44, 2.29) | 0.99 |
| VDRA | 1.53 (0.72, 3.26) | 0.27 |
| Calcium-based phosphate binders | 2.94 (1.55, 5.58) | 0.001 |
| Calcimimetics | 1.14 (0.61, 2.13) | 0.69 |
| Corrected Calcium | 4.01 (2.42, 6.67) | <0.001 |
| Phosphorus | 1.12 (0.95, 1.33) | 0.18 |
| Log-transformed intact PTH | 0.83 (0.54, 1.30) | 0.42 |
| Albumin | 1.11 (0.55, 2.25) | 0.78 |

*Due to the scarcity of hypercalcaemia events in the TPTD group (only one episode), 10 patients (TPTD users) were excluded.

HR, hazard ratio; CI, confidence interval; BP, bisphosphonate; DMAB, denosumab; TPTD, teriparatide; ROMO, romosozumab; DM, diabetes mellitus; CVD, cardiovascular disease; VDRA, vitamin D receptor agonists; PTH, parathyroid hormone.
